# Dilated cardiomyopathy due to novel LMNA mutation: a case report

**DOI:** 10.3389/fcvm.2024.1422151

**Published:** 2024-10-01

**Authors:** Riddhi Patel, Raj Patel, Ekta Patel, Mehul Patel

**Affiliations:** ^1^ Lake Erie College of Osteopathic Medicine, Erie, PA, United States; ^2^ Lake Erie College of Osteopathic Medicine, Greensburg, PA, United States; ^3^ St. Bonaventure University, St. Bonaventure, NY, United States; ^4^ Premier Medical Group—Cardiology Division, Newburgh, NY, United States

**Keywords:** LMNA mutation, dilated cardiomyopathy, ICD, sudden cardiac death, case report

## Abstract

A case of a 44-year-old man presenting with a family history of LMNA mutation and cardiac symptoms (dizziness, weakness, palpitations, and shortness of breath) congruent with dilated cardiomyopathy. Genetic testing revealed a novel likely pathogenic mutation of the LMNA gene (c.513G>A, exon 2) not previously associated with dilated cardiomyopathy, and the patient underwent guideline direct treatment for dilated cardiomyopathy. In patients with LMNA mutations, VTA risk should be calculated to determine the need for prophylactic ICD placement.

## Introduction

Causes of cardiomyopathy include ischemia, genetic aberrancies, infections, hypertension, alcoholism, and congenital defects. Genetic mutations resulting in hypertrophic cardiomyopathy are predominantly due to sarcomere mutations, whereas mutations in cytoskeletal proteins such as titin and lamins cause dilated cardiomyopathy (1, 2). LMNA mutations can result in various phenotypes including severe cardiac pathologies ranging from arrythmias to sudden cardiac death (3, 4). Identification of LMNA mutations in the familial line warrants genetic testing and cardiac evaluation of family members for adequate management. The LMNA, exon 2, c.513G>A mutation has not previously been associated with dilated cardiomyopathy and is not present in population databases. The discovery and reporting of this new LMNA mutation resulting in dilated cardiomyopathy is of great utility as providers become aware of this mutation and can more effectively manage patients.

## Case presentation

A 44-year-old male with no significant past medical history and family history positive for LMNA gene mutation in his maternal line presented to the clinic for cardiology evaluation with the complaint of chest tightness and associated dizziness and weakness. The patient denied any syncope or pedal edema. The patient's mother has cardiac manifestations of LMNA gene mutation consisting of atrial fibrillation (Afib) and left ventricular ejection fraction (LVEF) <35%, resulting in pacemaker/implantable cardioverter defibrillator (ICD) placement in her 40s. The patient was noted to have bradycardia and complaints of palpitations, dizziness, and shortness of breath. A Holter study, echocardiogram, carotid Doppler study, and exercise stress test were ordered for evaluation of symptoms. The physical exam was unremarkable.

The initial differential diagnoses for the etiology of the patient's symptoms included ischemic and nonischemic causes such as coronary artery disease, post-viral infection cardiomyopathy, alcohol-induced cardiomyopathy, and familial dilated cardiomyopathy.

**Figure 1:**
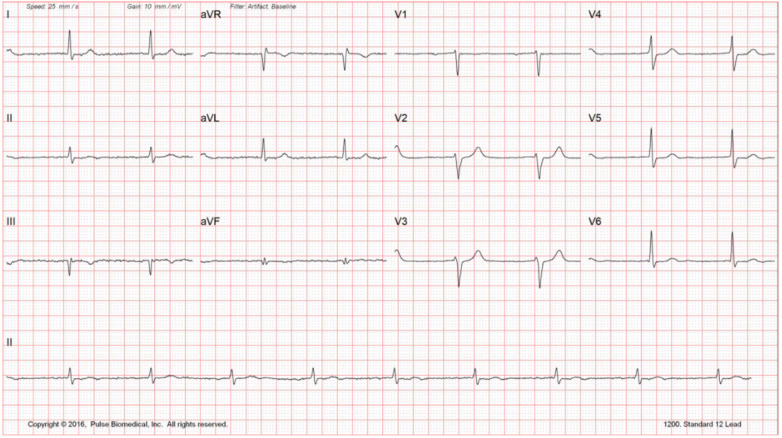
Resting EKG.

**Figure 2:**
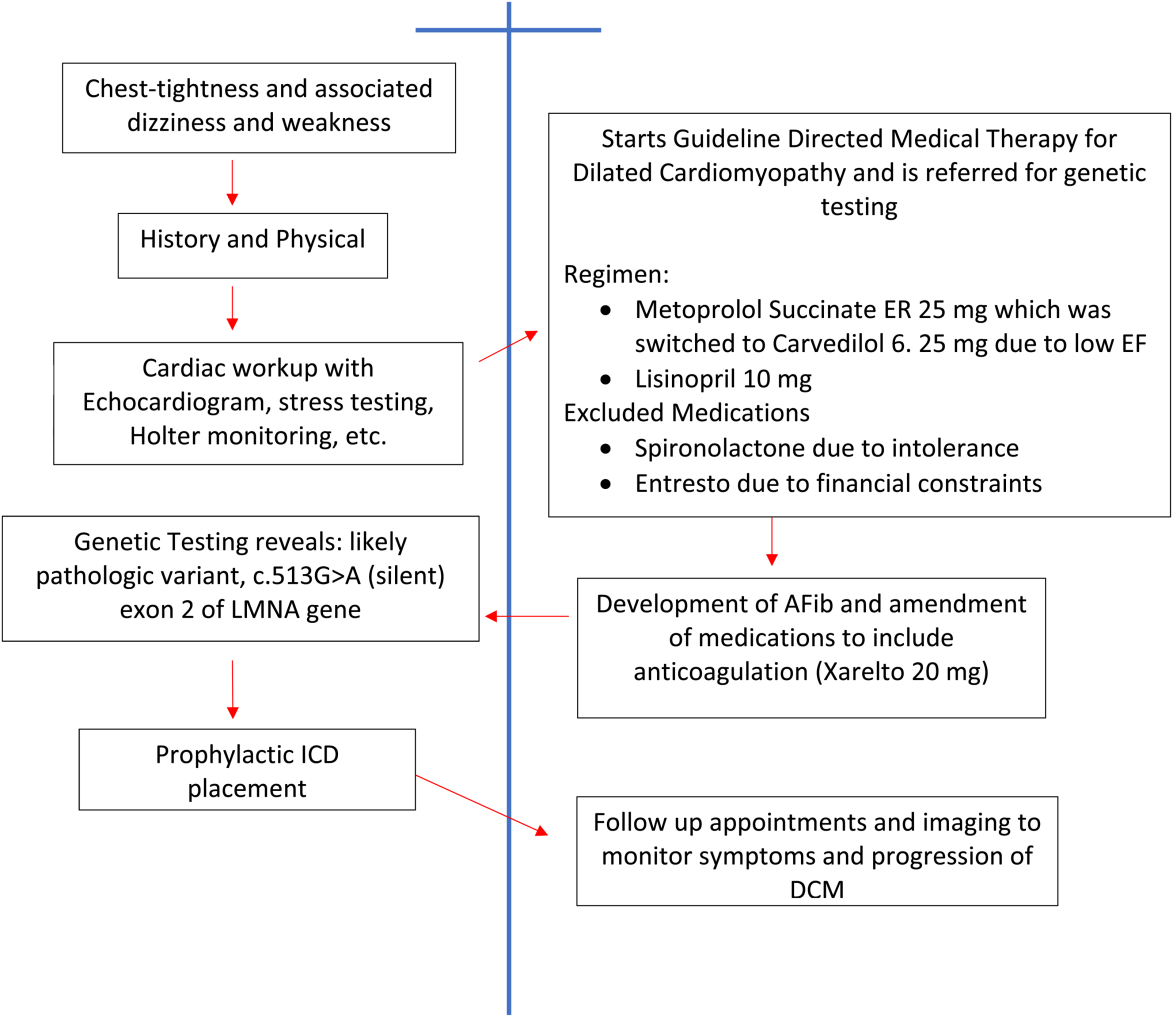
Timeline depicting medical management.

Routine laboratory testing did not reveal any significant findings (Figure 1). Echocardiogram reveals reduced EF at 35%–40% with mildly dilated left ventricle and global hypokinesis. Exercise stress testing was negative for stress-induced ischemia; atrial premature contractions with right bundle branch block pattern aberrancy were noted. Holter monitoring did not reveal any significant bradyarrhythmias or tachyarrhythmias. Carotid Doppler was negative for hemodynamically significant stenosis. The constellation of findings was highly suggestive of nonischemic cardiomyopathy. The patient was sent for electrophysiology consultation and genetic testing (Figure 2).

Guideline Directed Medical Therapy for the management of dilated cardiomyopathy was initiated, which included metoprolol succinate ER 25 mg, which was then switched to carvedilol 6.25 mg due to low EF, and lisinopril 10 mg. Spironolactone was not included in therapy due to medication intolerance and Entresto (sacubitril/valsartan) was not included due to financial constraints. Additionally, due to financial constraints the patient was unable to obtain genetic testing (Figure 2).

Approximately seven months after the initial presentation, the patient sustained a thirteen-hour period of Afib, with associated dizziness and anxiety. Echocardiography did not show worsening EF. The patient's medication regimen was now amended to include a direct oral anticoagulant (Xarelto 20 mg) due to the new onset of paroxysmal Afib (Figure 2).

Fifteen months after the initial presentation, genetic testing revealed a novel mutation in the sequencing of exon 2 of the LMNA gene. The mutation is likely a pathologic variant, c.513G>A (silent). This is the same mutation that was noted on the patient's mother's genetic testing. This mutation, although previously associated with other autosomal dominant laminopathies, has not been associated with dilated cardiomyopathy. The case was discussed with an electrophysiologist and the rest of the medical team, and the patient underwent prophylactic ICD placement for the LMNA laminopathy (Figure 2).

Although the clinical progression of the patient was only significant for symptomatic bradycardia and did not have ventricular tachyarrhythmia (VTA), an ICD was considered due to the high incidence of SCD in patients with laminopathies. In addition, due to the novel mutation, genetic testing and cardiac evaluation were recommended for all family members. The patient's daughters underwent genetic testing to determine whether they inherited the likely pathogenic variant. Genetic testing revealed that one of the daughters inherited the variant, and therefore is following up with a pediatric cardiologist for further evaluation and management.

## Discussion

The LMNA gene encodes two cytoskeletal nuclear proteins, Lamin A and C, both of which are essential for proper cardiac function. Lamins have a role in the process of gene expression and are implicated in the replication of DNA, regulation of the cell cycle, stabilization of the nucleus, and signal transduction processes, as well as many others (3, 4). Mutations in the LMNA gene have many implications leading to various manifestations of autosomal dominant and recessive conditions such as dilated cardiomyopathy, Emery-Dreifuss muscular dystrophy, congenital muscular dystrophy, Charcot Marie Tooth Disease, and Hutchinson-Gilford progeria syndrome (4). There are multiple families of lamins, but the Type A filament subtype is implicated in cardiac disease. Mutations in Type A filaments lead to mutations in lamin A/C, both of which result from alternative splicing of the LMNA gene. Mutations in lamin A/C result in nuclear instability, impaired signaling, and DNA replication, and the allelic heterozygosity allows for a variety of different implications in the structure and function of these proteins, leading to the pleomorphic presentation of the mutations (5). The most common manifestation of LMNA mutations are cardiolaminopathies due to alterations of myocardial cytoskeletal proteins, which manifests as cardiac dilation, conduction abnormalities, and arrhythmias (3, 4).

LMNA-cardiomyopathy is associated with 165 different mutations (4). Most pathogenic mutations are missense and nonsense mutations; however, other types of mutations have been identified as pathogenic as well. Mutations that result in shorter sequences due to aberrant splicing have been associated with an earlier incidence of arrhythmias and a decrease in EF (5). However, there is phenotypic discordance in individuals with identical mutations in the LMNA gene which may be due to influences by the environment or modifying genes. The phenotypic discordance can be attributed to the age-dependent penetrance seen in cardiolaminopathies. Initially, arrythmias may be noted, but as the patient gets older, there will be full penetrance with dilated cardiomyopathy and sudden cardiac death (5). The onset of arrhythmias usually ensues within the 3rd and 4th decades of life and full penetrance is most commonly seen by the 7th decade of life. A carrier of the LMNA mutation is at increased risk of sudden cardiac death before full penetrance of the mutation (4, 5).

The LMNA mutation seen in this patient has not yet been identified in literature and has shown to be a likely pathogenic mutation as evidenced by the patient's symptoms. Dilated cardiomyopathy is one of the leading causes of SCD, and prediction of SCD is challenging. However, there are certain symptoms and characteristics of dilated cardiomyopathy that increase the risk of SCD such as LVEF < 35%, NYHA class II or III heart failure, male sex, advanced age, smoking, diabetes, hypertension, and hypercholesterolemia (3, 6). Currently, treatment for dilated cardiomyopathy involves ICD placement for primary prevention of SCD if LVEF < 35% and NYHA class II or III symptoms on chronic guideline-directed medical therapy (7). Prophylactic ICD placement in patients with LMNA dilated cardiomyopathy is of great utility as these patients are at increased risk of VTAs, conduction abnormalities such as AV block, and SCD. Prediction of life-threatening ventricular tachyarrhythmia is determined with the LMNA-risk VTA calculator which considers the patients’ sex, the type of mutation the patient has (non-missense), whether the patient has an AV block, the presence of a non-sustained ventricular tachycardia, and LVEF (6, 8). The patient had 4 of the 5 risk factors included in the calculation of risk of SCD, as the patient did not have an event of non-sustained ventricular tachycardia. Using the LMNA-risk VTA calculator, the patient was determined to have a 33.9% 5-year risk of a life-threatening ventricular tachyarrhythmia. Therefore, the placement of a prophylactic ICD was indicated for the primary prevention of SCD.

LMNA mutations carry a poor prognosis, as cardiolaminopathies are associated with increased risk of arrythmias, sudden cardiac death, and worsening heart failure as well as increased need for cardiac transplantation due to rapid disease progression (9). There is limited data displaying an improvement in cardiac function with treatment. 5 years after diagnosis there is a 40% chance of mortality and 45% chance of SCD (4), and approximately 70% of patients develop an adverse cardiac event because of this cardiolaminopathy (5). With age, there is worsening of structural and electrical cardiac function in patients with LMNA cardiolaminopathy as evidenced by worsening right and left ventricular systolic dysfunction, right and left ventricular dilation, mitral regurgitation, tricuspid regurgitation, and conduction delays. These structural and electrical changes in patients with LMNA mutations results in worsening cardiac function and increased mortality (10).

## Conclusion

In conclusion, LMNA mutations may be a rare cause of DCM and the initial presenting symptom may be aberrancy in the conduction system resulting in arrythmias, therefore, a comprehensive screening with the inclusion of a comprehensive history and physical, laboratory, and cardiovascular assessment should be conducted to rule out other causes of arrythmias and aid in the diagnosis of a genetic cause. Consideration of cardiolaminopathies in the work up of a new onset arrhythmia can facilitate the optimization of treatment in these patients with prophylactic ICD placements.

## Data Availability

The raw data supporting the conclusions of this article will be made available by the authors, without undue reservation.
